# New Pessaries

**Published:** 1877-11-20

**Authors:** 


New Pessaries, by E. C. Gehrung, M. D., St. Louis, Mo. A pamphlet describing, with illustrations, a new Pessary for Antiflexion. A modified Retroversion Pessary, and a new Retroflexion Pessary.

Interesting, instructive and practical.

